# Commonly used software tools produce conflicting and overly-optimistic AUPRC values

**DOI:** 10.1101/2024.02.02.578654

**Published:** 2024-02-07

**Authors:** Wenyu Chen, Chen Miao, Zhenghao Zhang, Cathy Sin-Hang Fung, Ran Wang, Yizhen Chen, Yan Qian, Lixin Cheng, Kevin Y. Yip, Stephen Kwok-Wing Tsui, Qin Cao

**Affiliations:** 1 School of Biomedical Sciences, The Chinese University of Hong Kong, Shatin, New Territories, Hong Kong SAR, China; 2 Department of Computer Science and Engineering, The Chinese University of Hong Kong, Shatin, New Territories, Hong Kong SAR, China; 3 The First Affiliated Hospital, Sun Yat-sen University, Guangzhou, China; 4 Shenzhen People’s Hospital, First Affiliated Hospital of Southern University of Science and Technology, Second Clinical Medicine College of Jinan University, Shenzhen, China; 5 Sanford Burnham Prebys Medical Discovery Institute, La Jolla, CA, USA; 6 Hong Kong Bioinformatics Centre, The Chinese University of Hong Kong, Shatin, New Territories, Hong Kong SAR, China; 7 Shenzhen Research Institute, The Chinese University of Hong Kong, Shenzhen, China

## Abstract

The precision-recall curve (PRC) and the area under it (AUPRC) are useful for quantifying classification performance. They are commonly used in situations with imbalanced classes, such as cancer diagnosis and cell type annotation. We evaluated 10 popular tools for plotting PRC and computing AUPRC, which were collectively used in >3,000 published studies. We found the AUPRC values computed by the tools rank classifiers differently and some tools produce overly-optimistic results.

## Introduction

Many problems in computational biology can be formulated as binary classification, in which the goal is to infer whether an entity (e.g., a cell) belongs to a target class (e.g., a cell type). Accuracy, precision, sensitivity (i.e., recall), specificity, and F1 score (Supplementary Figure 1) are some of the measures commonly used to quantify classification performance, but they all require a threshold of the classification score to assign every entity to either the target class or not. The receiver operating characteristic (ROC) and precision-recall curve (PRC) avoid this problem by considering multiple thresholds [1], which allows detailed examination of the trade-off between identifying entities of the target class and wrongly including entities not of this class. It is common to summarize these curves by the area under them (AUROC and AUPRC, respectively), which is a value between 0 and 1, with a larger value corresponding to better classification performance.

When the different classes have imbalanced sizes (e.g., the target cell type has few cells), AUPRC is a more sensitive measure than AUROC [1–4], especially when there are errors among the top predictions (Supplementary Figure 2). As a result, AUPRC has been used in a variety of applications, such as reconstructing biological networks [5], identifying cancer genes [6] and essential genes [7], determining protein binding sites [8], imputing sparse experimental data [9], and predicting patient treatment response [10]. AUPRC has also been extensively used as a performance measure in benchmarking studies, such as the ones for comparing methods for analyzing differential gene expression [11], identifying gene regulatory interactions [12], and inferring cell-cell communications [13] from single-cell RNA sequencing data.

Given the importance of PRC and AUPRC, we analyzed commonly used software tools and found that they produce contrasting results, some of which are overly-optimistic.

## Results

### Basics

For each entity, a classifier outputs a score to indicate how likely it belongs to the target (i.e., “positive”) class. Depending on the classifier, the score can be discrete (e.g., random forest) or continuous (e.g., artificial neural network). Using a threshold *t*, the classification scores can be turned into binary predictions by considering all entities with a score ≥ *t* as belonging to the positive class and all other entities as not. When these predictions are compared to the actual classes of the entities, precision is defined as the proportion of entities predicted to be positive that are actually positive, while recall is defined as the proportion of actually positive entities that are predicted to be positive (Supplementary Figure 1).

The PRC is a curve that shows how precision changes with recall. In the most common way to produce the PRC, each unique classification score observed is used as a threshold to compute a pair of precision and recall values, which forms an anchor point on the PRC. Adjacent anchor points are then connected to produce the PRC.

**Figure 1:**
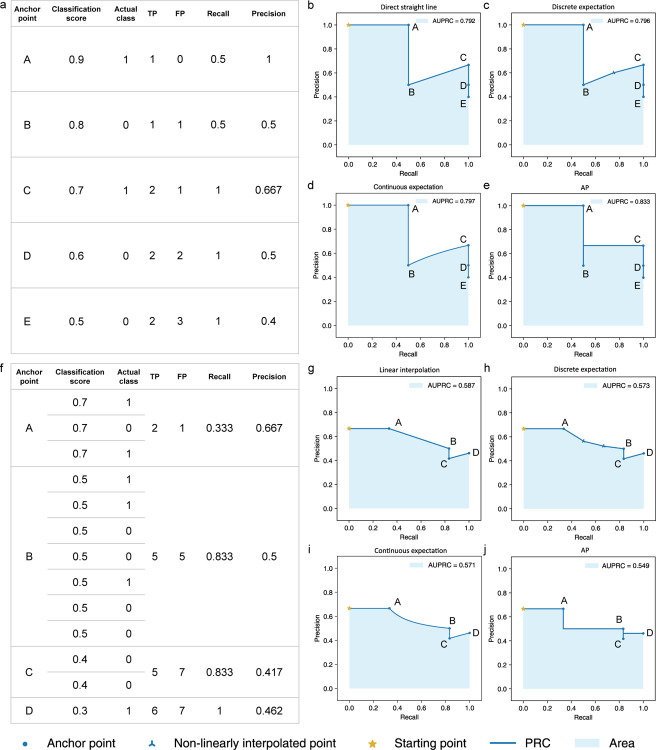
Different methods for connecting adjacent anchor points on the PRC. **a** An illustrative data set with no two entities receiving the same classification score. **b-e** Different methods for connecting adjacent anchor points when there are no ties in classification scores, namely **b** direct straight line, **c** discrete expectation, **d** continuous expectation, and **e** AP. f An illustrative data set with different entities receiving the same classification score. Each group of entities with the same classification score defines a single anchor point (A, B, C, and D, from 3, 7, 2, and 1 entities, respectively). **g-j** Different methods for connecting anchor point B to its previous anchor point, A, namely **g** linear interpolation, **h** discrete expectation, **i** continuous expectation, and **j** AP. In **c** and h, *tp* is set to 0.5 and 1 in Formula 1, respectively (Supplementary text).

When no two entities have the same score (Figure 1a), it is common to connect adjacent anchor points directly by a straight line [14–19] (Figure 1b). Another method uses an expectation formula, which we will explain below, to connect discrete points by piece-wise linear lines [20] (Figure 1c). The third method is to use the same expectation formula to produce a continuous curve between adjacent anchor points [17, 21] (Figure 1d). A fourth method that has gained popularity, known as Average Precision (AP), connects adjacent anchor points by step curves [15, 19, 22, 23] (Figure 1e). In all four cases, PRC estimates a function of precision in terms of recall based on the observed classification scores of the entities, and AUPRC estimates the integral of this function using trapezoids (in the direct straight line case), interpolation lines/curves (in the expectation cases), or rectangles (in the AP case).

When there are ties with multiple entities having the same score, which happens more easily with classifiers that produce discrete scores, these entities together define only one anchor point (Figure 1f). There are again four common methods for connecting such an anchor point to the previous anchor point, which correspond to the four methods for connecting anchor points when there are no ties (details in Supplementary text). The first method is to connect the two anchor points by a straight line [15, 18, 19] (Figure 1g). This method is known to easily produce overly-optimistic AUPRC values [2, 24], which we will explain below. The second method is to interpolate additional points between the two anchor points using a non-linear function and then connect the points by straight lines [14, 17, 20] (Figure 1h). The interpolated points appear at their expected coordinates under the assumption that all possible orders of the entities with the same score have equal probability. The third method uses the same interpolation formula as the second method but instead of creating a finite number of interpolated points, it connects the two anchor points by a continuous curve [17, 21] (Figure 1i). Finally, the fourth method comes naturally from the AP approach, which uses step curves to connect the anchor points [15, 19, 22, 23] (Figure 1j).

Using the four methods to connect anchor points when there are no ties and the four methods when there are ties can lead to very different AUPRC values (Figure 1, Supplementary Figure 3, and Supplementary text).

### Conceptual and implementation issues of some popular software tools

We analyzed 10 tools commonly used to produce PRC and AUPRC (Supplementary Table 1). Based on citations and keywords, we estimated that these tools have been used in >3,000 published studies in total (Methods).

The 10 tools use different methods to connect anchor points on the PRC and therefore they can produce different AUPRC values (Table 1, Supplementary Figures 4–7, and Supplementary text). As a comparison, all 10 tools can also compute AUROC, and we found most of them to produce identical values (Supplementary text).

We found five conceptual issues with some of these tools when computing AUPRC values (Table 1):

① Using the linear interpolation method to handle ties, which can produce overly-optimistic AUPRC values [2, 24]. When interpolating between two anchor points, linear interpolation produces higher AUPRC than the other three methods under conditions that can easily happen in real situations (Supplementary text)
② Always using (0, 1) as the starting point of the PRC (procedurally produced or conceptually derived, same for ③ and ⑤ below), which is inconsistent with the concepts behind the AP and non-linear expectation methods when the first anchor point with a non-zero recall does not have a precision of one (Supplementary text)
③ Not producing a complete PRC that covers the full range of recall values from zero to one
④ Ordering entities with the same classification score by their order in the input and then handling them as if they have distinct classification scores
⑤ Not putting all anchor points on the PRC

These issues can lead to overly-optimistic AUPRC values or change the order of two AUPRC values (Supplementary text, Supplementary Figures 8–13).

Some of these tools also produce a visualization of the PRC. We found three types of issues with these visualizations (Table 1):

- Producing a visualization of PRC that has the same issue(s) as in the calculation of AUPRC
- Producing a PRC visualization that does not always start the curve at a point with zero recall
- Producing a PRC visualization that always starts at (0, 1)

Finally, we also found some programming bugs and noticed that some tools require special attention for correct usage (both marked by ⚠ in Table 1).

### Inconsistent AUPRC values and contrasting classifier ranks produced by the popular tools

To see how the use of different methods by the 10 tools and their other issues affect PRC analysis in practice, we applied them to evaluate classifiers in four realistic scenarios.

**Figure 2:**
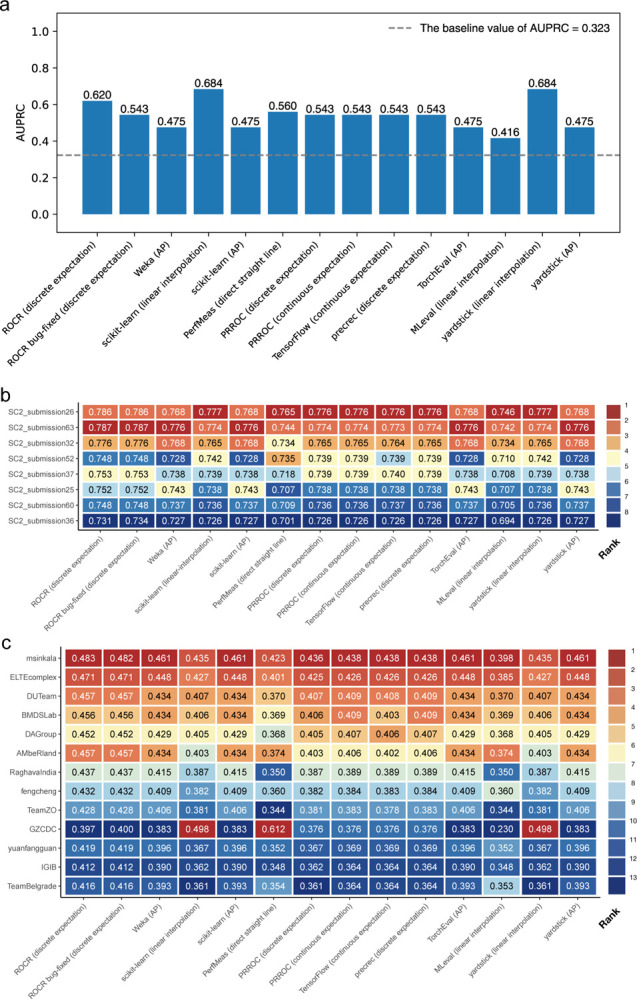
The AUPRC values computed by the 10 tools in several realistic scenarios. **a** Predicting CD4^+^ T cells from single-cell transcriptomic data. **b** Predicting inflammatory bowel disease cases that belong to the Ulcerative Colitis subtype in the sbv IMPROVER Metagenomics Diagnosis for Inflammatory Bowel Disease Challenge. Only the top 8 submissions according to PRROC (discrete expectation) AUPRC values are included. **c** Predicting cases with preterm prelabor rupture of membranes in the DREAM Preterm Birth Prediction Challenge. In **b** and **c**, each entry shows the AUPRC value and the background color indicates its rank among the competitors.

In the first scenario, we analyzed data from a COVID-19 study [25] in which patient blood samples were subjected to Cellular Indexing of Transcriptomes and Epitopes by Sequencing (CITE-seq) assays [26]. We constructed a classifier for predicting CD4^+^ T cells, which groups the cells based on their transcriptome data alone and assigns a single cell type label to each group. Using cell type labels defined by the original authors as reference, which were obtained using both antibody-derived tags (ADTs) and transcriptome data, we computed the AUPRC of the classifier. Figure 2a shows that the 10 tools produced 6 different AUPRC values, ranging from 0.416 to 0.684. In line with the conceptual discussions above, the AP method generally produced the smallest AUPRC values while the linear interpolation method generally produced the largest, although individual issues of the tools created additional variations of the AUPRC values computed.

In the second scenario, we compared the performance of different classifiers that predict whether a patient has the Ulcerative Colitis (UC) subtype of inflammatory bowel disease (IBD) or does not have IBD, based on metagenomic data (processed taxonomy-based profile) [27]. The predictions made by these classifiers were submitted to the sbv IMPROVER Metagenomics Diagnosis for IBD Challenge. Their performance was determined by comparing against diagnosis of these patients based on clinical, endoscopic, and histological criteria. Figure 2b shows that based on the AUPRC values computed, the 10 tools ranked the classifiers differently. For example, among the top 8 submissions with the highest performance, the classifier in submission 26 was ranked first in 8 cases, sole second place in 2 cases, and tied second place with another classifier in 4 cases (Figure 2b and Supplementary Figure 14). We observed similar rank flips when considering the top 30 submissions (Supplementary Figures 15 and 16).

In the third scenario, we compared the performance of different classifiers in identifying preterm prelabor rupture of the membranes (PPROM) cases from normal pregnancy in the DREAM Preterm Birth Prediction Challenge [28]. Based on the AUPRC values produced by the 10 tools, the 13 participating teams were ranked very differently (Figure 2c and Supplementary Figure 17). For example, Team “GZCDC” was ranked first (i.e, highest) in 3 cases, tenth in 4 cases, and thirteenth (i.e., lowest) in 7 cases. In addition to differences in the ranks, some of the AUPRC values themselves are also very different. For example, the AUPRC values computed by PerfMeas and MLeval have a Pearson correlation of −0.759, which shows that their evaluations of the 13 teams were almost completely opposite.

In the fourth scenario, we compared 29 classifiers that predicted target genes of transcription factors in the DREAM5 challenge [29]. Again, some classifiers received very different ranking based on the AUPRC values computed by the different tools (Supplementary Figures 18 and 19). For example, the classifier named “Other4” was ranked second based on the AUPRC values computed by PerfMeas but it was ranked twenty-fifth based on the AUPRC values computed by MLeval. In general, tools that use the discrete expectation, continuous expectation, and AP methods are in good agreements in this scenario, but they differ substantially from tools that use the linear interpolation method.

## Conclusions

Due to their highly technical nature, it is easy to overlook the inconsistencies and issues of the software tools used for producing PRC and AUPRC. Some possible consequences include reporting overly-optimistic AUPRC, ranking classifiers differently by different tools, and introducing biases to the evaluation process, such as inflating the AUPRC of classifiers that produce discrete scores.

## Methods

### Information about the tools

In this study, we included 12 tools commonly used for PRC and ROC analyses (Supplementary Table 1). For each tool, we analyzed the latest stable version of it as of August 15, 2023. Because TorchEval had not released a stable version, we analyzed the latest version of it, version 0.0.6. Among the 12 tools, ten can compute both AUROC and AUPRC, while the remaining two can only compute AUROC. We focused on these 10 tools in the study of PRC and AUPRC. Some tools provide multiple methods for computing AUROC/AUPRC.

For tools with an associated publication, we obtained its citation count from Google Scholar. If a tool has multiple associated publications, we selected the one with the largest number of citations. As a result, the citation counts we report in Supplementary Table 1 are underestimates if different publications associated with the same tool are not always cited together.

The Comprehensive R Archive Network (CRAN) packages PerfMeas and MLeval did not have an associated formal publication but only release notes. In each of these cases, we used the package name as keyword to search on Google Scholar and then manually checked the publications returned to determine the number of publications that cited these packages.

The CRAN package yardstick also did not have an associated formal publication. However, we were not able to use the same strategy as PerMeas and MLeval to determine the number of publications that cited the yardstick package since “yardstick” is an English word and the search returned too many publications to be verified manually. Therefore, we only counted the number of publications that cited yardstick’s release note, which is likely an underestimate of the number of publications that cited yardstick.

All citation counts were collected on October 9, 2023.

For tools with an associated formal publication, based on our collected lists of publications citing the tools, we further estimated the number of times the tools were actually used in the studies by performing keyword-based filtering. Specifically, if the main text or figure captions of a publication contains either one of the keywords “AUC” and “AUROC”, we assumed that the tool was used in that published study to perform ROC analysis. In the case of PRC, we performed filtering in two different ways and reported both sets of results in Supplementary Table 1. In the first way, we assumed a tool was used in a published study if the main text or figure captions of the publication contains any one of the following keywords: “AUPR”, “AU-PR”, “AUPRC”, “AU-PRC”, “AUCPR”, “AUC-PR”, “PRAUC”, “PR-AUC”, “area under the precision recall”, and “area under precision recall”. In the second way, we assumed a tool was used in a published study if the main text or figure captions of the publication contains both “area under” and “precision recall”.

For the CRAN packages PerfMeas and MLeval, we estimated the number of published studies that actually used them by searching Google Scholar using the above three keyword sets each with the package name appended. We found that for all the publications we considered as using the packages in this way, they were also on our lists of publications that cite these packages. We used the same strategy to identify published studies that used the CRAN package yardstick. We found that some of these publications were not on our original list of publications that cite yardstick, and therefore we added them to the list and updated the citation count accordingly.

TorchEval was officially embedded into PyTorch in 2022. Due to its short history, among the publications that cite the PyTorch publication, we could not find any of them that used the TorchEval library.

### Data collection and processing

We used four realistic scenarios to illustrate the issues of the AUPRC calculations.

In the first scenario, we downloaded CITE-seq data produced from COVID-19 patient blood samples by the COVID-19 Multi-Omic Blood ATlat (COMBAT) consortium [25]. We downloaded the data from https://doi.org/10.5281/zenodo.6120249 and used the data in the “COMBAT-CITESeq-DATA” archive in this study. We then used a standard procedure to cluster the cells based on the transcriptome data and identified CD4^+^ T cells. Specifically, we extracted the raw count matrix of the transcriptome data and ADT features (“X” object) and the annotation data frame (“obs” object) from the H5AD file. We dropped all ADT features (features with names starting with “AB-”) and put the transcriptome data along with the annotation data frame into Seurat (version 4.1.1). We then log-normalized the transcriptome data (method “NormalizeData()”, default parameters), identified highly-variable genes (method “FindVariableFeatures()”, number of variable genes set to 10,000), scaled the data (method “ScaleData()”, default parameters), performed principal component analysis (method “RunPCA()”, number of principal components set to 50), constructed the shared/knearest neighbor (SNN/kNN) graph (method “FindNeighbours()”, default parameters), and performed Louvain clustering of the cells (method “FindClusters()”, default parameters). We then extracted the clustering labels generated and concatenated them with cell type, major subtype, and minor subtype annotations provided by the original authors, which were manually curated using both ADT and transcriptome information.

Our procedure produced 29 clusters, which contained 836,148 cells in total. To mimic a classifier that predicts CD4^+^ T cells using the transcriptome data alone, we selected one cluster and “predicted” all cells in it as CD4^+^ T cells and all cells in the other 28 clusters as not, based on which we computed an AUPRC value by comparing these “predictions” with the original authors’ annotations. We repeated this process for each of the 29 clusters in turn, and chose the one that gave the highest AUPRC as the final cluster of predicted CD4^+^ T cells.

For the second scenario, we obtained the data set used in the sbv IMPROVER (Systems Biology Verification combined with Industrial Methodology for PROcess VErification in Research) challenge on inflammatory bowel disease diagnosis based on metagenomics data [27]. The challenge involved 12 different tasks, and we focused on the task of identifying UC samples from non-IBD samples using the processed taxonomy-based profile as features. The data set contained 32 UC samples and 42 non-IBD samples, and therefore the baseline AUPRC was $\frac{32}{32+42}=0.432$. There were 60 submissions in total, which used a variety of classifiers. We obtained the classification scores in the submissions from Supplementary Information 4 of the original publication [27]. When we extracted the classification scores of each submission, we put the actual positive entities before the actual negative entities. This ordering did not affect the AUPRC calculations of most tools except those of PerfMeas, which depend on the input order of the entities with the same classification score.

To see how the different tools rank the top submissions, we first computed the AUPRC of each submission using PRROC (option that uses the discrete expectation method to handle ties) since we did not find any issues with its AUPRC calculations (Table 1). We then analyzed the AUPRC values produced by the 10 tools based on either the top 8 (Figure 2b and Supplementary Figure 14) or top 30 (Supplementary Figures 15 and 16) submissions.

For the third scenario, we downloaded the data set used in the Dialogue on Reverse Engineering Assessment and Methods (DREAM) Preterm Birth Prediction Challenge [28] from https://www.synapse.org/#!Synapse:syn22127152. We collected the classification scores, from the object “prpile” in each team’s RData file, and the actual classes produced based on clinical evidence, from “anoSC2_v21_withkey.RData” (https://www.synapse.org/#!Synapse:syn22127343). The challenge contained 7 scenarios, each of which had 2 binary classification tasks. For each scenario, 10 different partitioning of the data into training and testing sets were provided. We focused on the task of identifying PPROM cases from the controls under the D2 scenario defined by the challenge. For this task, the baseline AUPRC value averaged across the 10 testing sets was 0.386. There were 13 participating teams in total. For each team, we extracted its classification scores and placed the actual positive entities before the actual negative entities. For submissions that contained negative classification scores, we re-scaled all the scores to the range between 0 and 1 without changing their order since TensorFlow expects all classification scores to be between zero and one (Supplementary text). Finally, for each team, we computed its AUPRC using each of the 10 testing sets and reported their average. We note that the results we obtained by using PRROC (option that uses the continuous expectation method to handle ties) were identical to those reported by the challenge organizer.

For the fourth scenario, we obtained the data set used in the DREAM5 challenge on reconstructing transcription factor-target networks based on gene expression data [29]. The challenge included multiple networks and we focused on the *E. coli in silico* Network 1, which has a structure that corresponds to the real E. coli transcriptional regulatory network [29]. We obtained the data from Supplementary Data of the original publication [29]. There were 29 submissions in total. For each submission, we extracted the classification scores of the predicted node pairs (each pair involves one potential transcription factor and one gene it potentially regulates) from Supplementary Data 4 and compared them with the actual classes (positive if the transcription factor actually regulates the gene; negative if not) in the gold-standard network from Supplementary Data 3. Both the submissions and the gold-standard were not required to include all node pairs. To handle this, we excluded all node pairs in a submission that were not included in the gold-standard (because we could not judge whether they are actual positives or actual negatives), and assigned a classification score of 0 to all node pairs in the gold-standard that were not included in a submission (because the submission did not give a classification score to them). The gold-standard contained 4,012 interacting node pairs and 274,380 non-interacting node pairs, and therefore the baseline AUPRC value was $\frac{4012}{4012+274380}=0.014$.

## Supplementary Material

1

## Figures and Tables

**Table 1:** Methods used by the different software tools to connect anchor points and issues found in their calculation of AUPRC and construction of the PRC. For tools that can connect anchor points in multiple ways, we show each of them in a separate row. The AUPRC and PRC issues are defined in the text and detailed in Supplementary text. “—” means no issues found.

Tool	Anchor points connection		AUPRC issues	PRC issues
	Without ties	With ties		
ROCR	Direct straight line	Discrete expectation	②⑤⚠	||
Weka	AP	AP	—	||
scikit-learn	Direct straight line	Linear interpolation	①②	(No visualization)
	AP	AP	—	|||
PerfMeas	Direct straight line	Direct straight line*	③④	|
PRROC	Direct straight line	Discrete expectation	—	⚠
	Continuous expectation	Continuous expectation	—	—
TensorFlow	Continuous expectation	Continuous expectation	⚠	(No visualization)
precrec	Discrete expectation	Discrete expectation	—	—
TorchEval	AP**	AP	—	(No visualization)
MLeval	Direct straight line	Linear interpolation	①③	|
yardstick	Direct straight line	Linear interpolation	①②	|
	AP	AP	—	(No visualization)

* PerfMeas orders entities with the same classification score by their order in the input and then defines anchor points as if there are no ties.

** The source code of TorchEval states that it uses Riemann integral to compute AUPRC, which is equivalent to AP.

## Data Availability

All data used in this study can be accessed following the procedure described in the “Data collection and processing” part of the Methods section.
